# Global initiative for congenital toxoplasmosis: an observational and international comparative clinical analysis

**DOI:** 10.1038/s41426-018-0164-4

**Published:** 2018-09-27

**Authors:** Kamal El Bissati, Pauline Levigne, Joseph Lykins, El Bachir Adlaoui, Amina Barkat, Amina Berraho, Majda Laboudi, Bouchra El Mansouri, Azeddine Ibrahimi, Mohamed Rhajaoui, Fred Quinn, Manoradhan Murugesan, Fouad Seghrouchni, Jorge Enrique Gómez-Marín, François Peyron, Rima McLeod

**Affiliations:** 1 Department of Ophthalmology and Visual Sciences, University of Chicago, Chicago, IL 60637 USA; 2 Institut de Parasitologie et de Mycologie Médicale Hôpital de la Croix Rousse, 103 grande rue de la Croix Rousse, 69317 Lyon, France; 3 Department of Emergency Medicine, Department of Internal Medicine, Virginia Commonwealth University Health System, Richmond, VA 23219 USA; 4 Institut National d’ Hygiène, Rabat, Morocco; 5 Research Team on Mother-Child Health and Nutrition, Faculté de Médecine et de Pharmacie de Rabat, Université Mohammed V, Rabat, Morocco; 6 Department d’Ophtalmologie, Hôpital des Spécialités, CHU, P6220 Rabat, Morocco; 7 Faculté de Médecine et de Pharmacie de Rabat, Université Mohammed V, Rabat, Morocco; 8 Department of Infectious Diseases, College of Veterinary Medicine, University of Georgia, Athens, GA 30602 USA; 9 Analytics, Graham School, University of Chicago, Chicago, IL 60637 USA; 10 Grupo de Estudio en Parasitología Molecular (GEPAMOL), Centro de Investigaciones Biomédicas, Universidad del Quindio, Av. Bolivar 12N, Armenia, Quindio Colombia; 11 Department of Ophthalmology and Visual Sciences, Department of Pediatrics (Infectious Diseases), Institute of Genomics, Genetics, and Systems Biology, Global Health Center, Toxoplasmosis Center, CHeSS, The College, University of Chicago, Chicago, IL 60637 USA

## Abstract

Globally, congenital toxoplasmosis remains a significant cause of morbidity and mortality, and outbreaks of infection with *T. gondii* represent a significant, emerging public health burden, especially in the developing world. This parasite is a threat to public health. Disease often is not recognized and is inadequately managed. Herein, we analyze the status of congenital toxoplasmosis in Morocco, Colombia, the United States, and France. We identify the unique challenges faced by each nation in the implementation of optimal approaches to congenital toxoplasmosis as a public health problem. We suggest that developed and developing countries use a multipronged approach, modeling their public health management protocols after those in France. We conclude that education, screening, appropriate treatment, and the development of novel modalities will be required to intervene successfully in caring for individuals with this infection. Gestational screening has been demonstrated to be cost-effective, morbidity-sparing, and life-saving. Recognition of the value and promise of public health interventions to prevent human suffering from this emerging infection will facilitate better patient and societal outcomes.

## Introduction

Toxoplasmosis, a disease caused by the Apicomplexan parasite *Toxoplasma gondii*, represents an emerging global public health threat. Toxoplasmosis causes significant morbidity and mortality globally^1^. It is particularly a problem for developing countries in warmer climates, where the public health infrastructure is less well-established and the highly resistant oocyst contaminates the environment longer^2^. While the disease has long been recognized, outbreaks of *T. gondii* infection are not uncommon, and their frequency and severity represent a significant public health burden^3–6^. Moreover, definitive feline hosts of the parasite are ubiquitous. Cats are capable of shedding hundreds of millions of oocysts over 2 weeks after primary infection, contaminating soil and water^3^. This water and soil contamination has been directly implicated in multiple epidemics^3,4,6^. *T. gondii* can also be contracted from the consumption of undercooked meat and vegetables contaminated with parasites^7^.

The parasite is capable of causing a range of clinical manifestations, from a self-limited, mild illness to chorioretinitis, meningoencephalitis, and devastating congenital infection^8^. This latter presentation is of particular importance, as vertical transmission represents a point for intervention to reduce suffering, morbidity, and mortality. Congenitally infected infants can present, dramatically, with hydrocephalus, epilepsy, and loss of sight^8^. However, the presentation can be more subtle and, with antenatal treatment, the disease can be mild or asymptomatic^8^. Recrudescence across time is common and presents a challenge to the management of patients infected with *T. gondii*^8^. Early identification and treatment of acutely infected pregnant women reduces rates of vertical transmission and disease severity in the affected fetus^9,10^. Additionally, mathematical models provide evidence that gestational screening and treatment could be cost-saving^11^. Moreover, an actual robust cost benefit has been shown in Austria^12^. Maternal infection with *Toxoplasma* can be asymptomatic, and exposure may go unrecognized^6^. Thus, serologic screening is the mainstay of identifying patients for treatment.

Diagnosis of congenital toxoplasmosis is made using clinical findings and laboratory testing for the pregnant woman, fetus, and infant, often together^8,13^. Prevention and treatment utilize anti-parasitic medications. Spiramycin concentrates in the placenta and blocks or delays transmission to the fetus, resulting in fewer infections or a milder infection. Pyrimethamine and sulfadiazine, with folinic acid, are the mainstays of treatment but do not treat the latent bradyzoite life-stage; thus, they are effective in active infection but not definitively curative^8^. Novel developments with respect to medications and vaccines will likely revolutionize prevention and treatment in the coming years^14,15^.

 Current treatment of confirmed fetal infection requires treatment with pyrimethamine, sulfadiazine, and folinic acid^8^. There was an unprecedented 5000-fold increase in the price of pyrimethamine in the US market recently, presenting a new challenge to the management of infected patients. New medicines that eliminate the encysted parasite are urgently needed in light of these challenges. New targets for drug designs include new approaches to DHFR, ENR, cytochrome B/C Q_i_, calcium kinase, and sodium ATPase^14,16–19^. These studies will likely revolutionize treatment in the coming years. Vaccines are also urgently needed to prevent *Toxoplasma* infection. Engineering recombinant proteins, immunosense approaches, and utilizing information learned from live attenuated vaccines will provide key information about priming the perfect protective immune response^15,20^.

 A real estimation of the incidence of congenital toxoplasmosis is difficult because, in the majority of countries, it is not a disease for which reporting of the diagnosis to public health officials is mandatory, and neither pre- nor postnatal screening programs have been implemented in many countries. Estimations that do exist are derived from prenatal or newborn screening programs or cross-sectional epidemiological studies. Additionally, even within a single country, wide variations in disease incidence can occur as a consequence of diverse natural geographic conditions between regions or due to temporal trends^21–23^. In France, where universal systematic screening of pregnant women was implemented in 1978, 272 cases were reported in 2007^24^, and the incidence of seroconversion during pregnancy in 2010 was estimated at 2 per 1000 susceptible pregnant women but has decreased markedly during the last 30 years^25^. This decrease may be explained by reduced exposure to the parasite as a result of changes in food habits and improved hygiene practices in meat production. In the United States, it is estimated that the incidence of congenital toxoplasmosis ranges from 500 to 5000 cases annually^11,26^. In Colombia (South America), a multicentric national screening of 15,000 newborns found an incidence of 1 case per 1000 newborns, with important regional variations related to mean annual rainfall^23^. The most important estimation about the global incidence of congenital toxoplasmosis was a meta-analysis published in 2013 in the Bulletin of the World Health Organization that described a global annual incidence of congenital toxoplasmosis of 190,100 cases (95% confidence interval, CI: 179,300–206,300). This was equivalent to a burden of 1.20 million disability-adjusted life years (DALYs) (95% CI: 0.76–1.90). High burdens were seen in South America and in some Middle Eastern and low-income countries^1^. Data for many other parts of the world are also available and merit a brief discussion. There is a paucity of evidence about seroprevalence in the general population in many Asian countries, although there is significant regional variability in known rates. East Asian countries, such as China, South Korea, and Japan, have comparatively lower rates of seroprevalence, often 10% or less^27–31^. A handful of studies demonstrate that screening for *T. gondii* infection does occur in approximately half of Japan’s obstetric facilities^32,33^. Seroprevalence rates are higher in Malaysia and Indonesia^34,35^. There is no evidence of routine screening in these locations. The Indian subcontinent demonstrates a high seroprevalence as well, with some reports indicating rates upto 40%^36^. In many of these countries, the prevalence of congenital toxoplasmosis is simply not reported nor recognized. Our literature search could not find any current data regarding the prevalence of seropositivity for *T. gondii* in Russia. A review of the global state of toxoplasmosis by Torgerson and Mastroiacovo described an increase in seroprevalence in Russia, although the magnitude of this increase was not discussed^1^. The WHO region containing Russia, as well as surrounding Eastern European states, was shown to have an incidence of congenital toxoplasmosis of 1.6 per 1000 live births, a high incidence. Kyrgyzstan, for example, had an estimated 173 seroconversion events to occur during gestation each year, and it was noted that the seroprevalence in rural areas was 5.1%, whereas the seroprevalence in urban areas was higher, at 16.4%^37^. The only recent study regarding congenital toxoplasmosis in Australia estimated a prevalence of 0.23 infected children per 1000 live births^38^. Melbourne had a seroprevalence of 23% in a group of pregnant women, with none being aware of a past infection, highlighting a relatively high prevalence with a large susceptible population that is unable to reliably identify past exposure to *T. gondii*^39^.

 Congenital toxoplasmosis is not an emerging infectious disease in the traditional sense of the term of being a disease that is increasing in frequency. However, it has characteristics that merit further consideration, and an expansive definition is in order to encompass this important parasitic infection. Its prevalence, for example, is not increasing, so far as the literature is able to demonstrate. In fact, the seroprevalence of antibodies against *T. gondii* is declining in some parts of the world^40,41^. However, it is very likely that our current understanding of congenital toxoplasmosis incidence and prevalence is a substantial underestimate, and enhanced recognition of the implications of this infection—for the affected individual, his or her family, and health systems—means that we are likely only seeing the tip of an iceberg that is congenital toxoplasmosis. Most patients with congenital toxoplasmosis in countries where systematic screening is not routine are diagnosed with severe disease at birth^42^. It has also been demonstrated that congenital toxoplasmosis can present without symptoms at birth but that this does not guarantee the absence of symptoms in the future; morbidity and even mortality can occur in such patients. Thus, the public health impact of this infection can be large, and the true magnitude of this problem cannot be accurately estimated in the absence of systematic screening. Costs for treatment are also enormous, with the burden disproportionately falling on developing countries. Climate change may impact the distribution of this parasite in the environment, as the parasite can persist in warm, moist soil or water for upto a year^43,44^. Changing conditions may promote oocyst persistence and, therefore, risk of infection. Moreover, the availability of rapid transportation on a global scale ensures that virulent parasite strains, once confined to the tropics, are no longer so restricted in their geographical range. Parasites with virulomes suggestive of South American lineage are observed in the United States^45^. This almost certainly occurs internationally as well. Therefore, with difficulty in the estimation of prevalence, substantial impact on maternal–child health as well as cost, the potential role of climate change and global transportation in the movement of parasite strains across international borders, congenital toxoplasmosis represents an important emerging infectious disease.

**Fig. 1:**
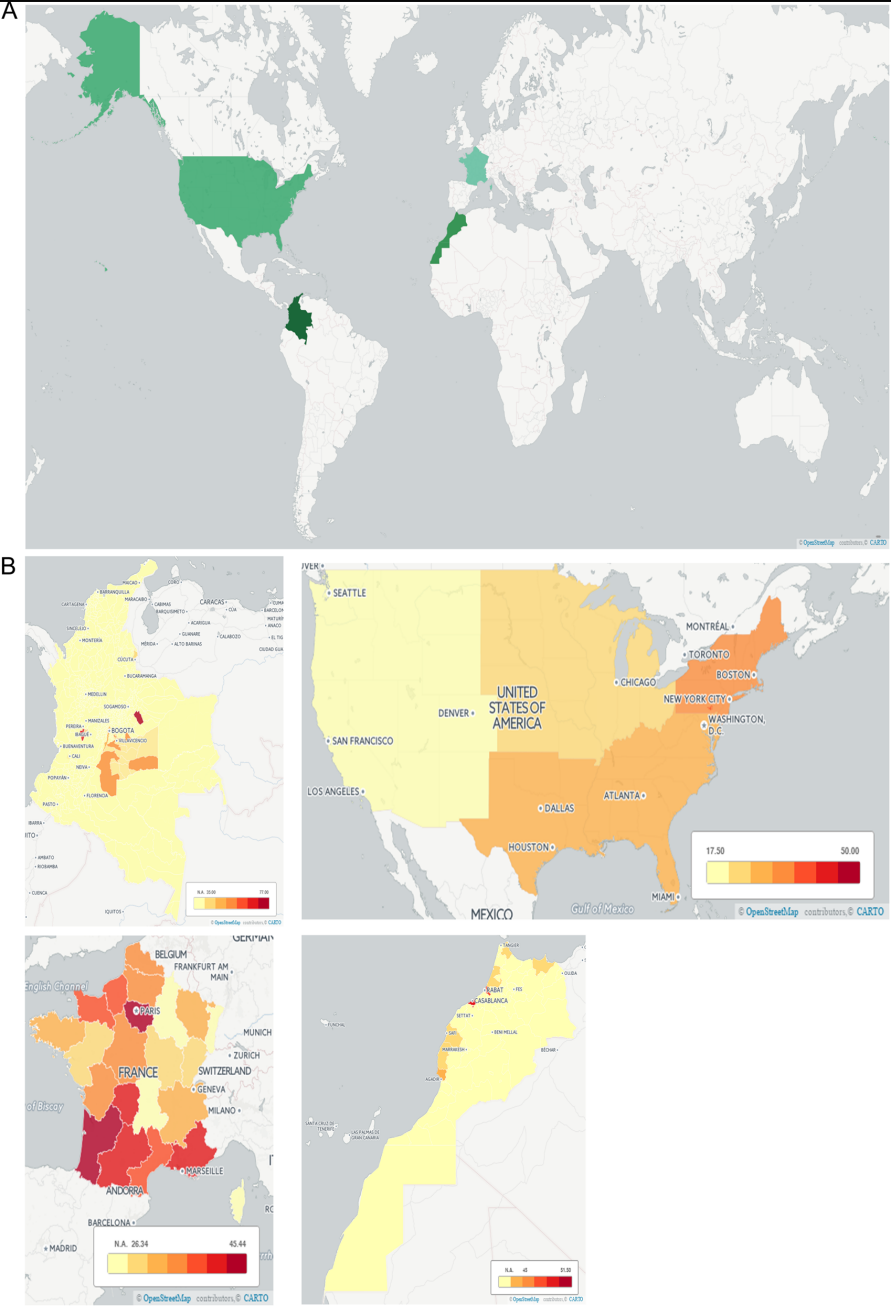
Seroprevalence of *T. gondii* across four countries. **a** Countries compared herein are highlighted. Across our countries of interest, there is significant variation in seroprevalence, with the United States having the lowest overall seroprevalence and Colombia having the highest. Contributing factors to this variation must include exposure to oocysts from cats and contaminated meat. Factors that influence these exposures include climate, host behaviors, and public health infrastructure. **b** There is regional variation within countries. In Colombia, there is significant variation that correlates with annual rainfall, which is associated with oocyst survival and with oocysts contaminating the environment^55^. Regions without seroprevalence data available are marked N.A. (no data available), while increasing rates of seroprevalence are indicated by darkening of the shading of the region. Seroprevalence data for the generation of these maps were obtained from several sources^40,41,47,49,55^. Maps were generated with Carto

Seroprevalence varies globally and within countries. For a presentation of this variation, see Fig. 1, where we compare individual experiences of nations in Europe, Africa, and North and South America with respect to this emerging infectious disease. In addition to providing a summary of present-day national approaches, we suggest several steps to develop successful programs for the management of toxoplasmosis at a national level, drawing on collective experience and evidence obtained over the last several decades.

## Country selection, search strategy, and selection criteria

Establishment of a rational approach to the management of *T. gondii* infection in Morocco was the subject of the 1st International Meeting on Congenital Toxoplasmosis in November 2016 in Rabat, Morocco, in partnership with the Moroccan Health Ministry. Prominently featured nations included Morocco, Colombia, the United States, and France. These countries, therefore, formed the basis of this analysis. International differences in approach, highlighted herein, lend themselves to a more generalized discussion of how best to prevent congenital infections such as toxoplasmosis.

References were identified via use of PubMed from 1990 to 2016 with use of the terms “congenital toxoplasmosis”, “Morocco”, “Colombia”, “United States”, and “France”. Particularly relevant articles, as well as those before 1990, were identified from the personal files of the authors. Google Scholar was also utilized for article identification. Additionally, articles cross-referenced in these articles were reviewed. Articles published in English, French, and Spanish were included.

## Country case studies

### Morocco

A North African country with a nominal GDP ranking of 60, Morocco represents a new frontier in studying congenital toxoplasmosis^46^. Previously, there was a paucity of data regarding disease epidemiology and prevalence, but new initiatives and research presently being established through the Ministry of Health and collaborations with the University of Chicago is leading to the recognition that toxoplasmosis is a significant public health challenge in the country. Morocco presents unique challenges to the full identification and reporting of persons with congenital infection and establishment of infrastructure for treatment after diagnosis. Morocco’s health policy is heavily influenced by French approaches. This is demonstrated in the present national mandate for gestational screening for *T. gondii* infection. However, the infrastructure does not facilitate follow-up, and medicine to treat infected mothers is often unavailable or only acquired at a substantial expense. At present, pregnant women are often screened only once, early in pregnancy and are told that they are seronegative or seropositive. Due to frequent misunderstanding of the implications of these results, women often do not present any further for additional screening. This situation, coupled with reports that clinicians predominantly order only *T. gondii* IgG and not the IgM needed to identify acute and, therefore, treatable infections, means that the present approach is suboptimal.

The few studies that have been conducted have reported a seroprevalence among pregnant women ranging in Rabat between 47 and 50.5%^47,48^, in Casablanca at 51.5%, and in the Safi-Essaouira region at 45%^49^. Nador, in northern Morocco, had a seroprevalence of 34.3%, whereas Tétouan and Kenitra had seroprevalences of 42% and 37.7%, respectively^49^. In the south, the seroprevalence in Agadir-Inezgane was 47.33%^50^. Early data indicate an impact of the oceanic climate, with an increasing prevalence of toxoplasmosis in this region. Despite regional variation in seroprevalence, rates found in this North African nation are substantially higher than those found in many areas of the United States. A seroprevalence of 50% indicates a high at-risk population (50%) and a high exposure rate, increasing the risk of infection during gestation. Thus, the current situation in Morocco represents a dangerous combination of a large at-risk population with significant environmental contamination and inadequate infrastructure for prompt diagnosis and treatment of acutely infected pregnant women.

The seroprevalence in Morocco does not vary greatly from reported rates in neighboring Algeria (47.8%) and Tunisia (47.7%)^51,52^. This finding is possibly attributable to similar culinary and cultural practices shared by these Maghreb countries. Most infections in Morocco are believed to be secondary to oocyst contamination of soil^48^. Eating undercooked meat and keeping cats do not seem to be primary causes for infection transmission in the country according to epidemiological survey data. These latter risk factors have been noted in French and US studies^53,54^. Outbreaks attributed to contaminated meat or water have not been recognized.

**Fig. 2:**
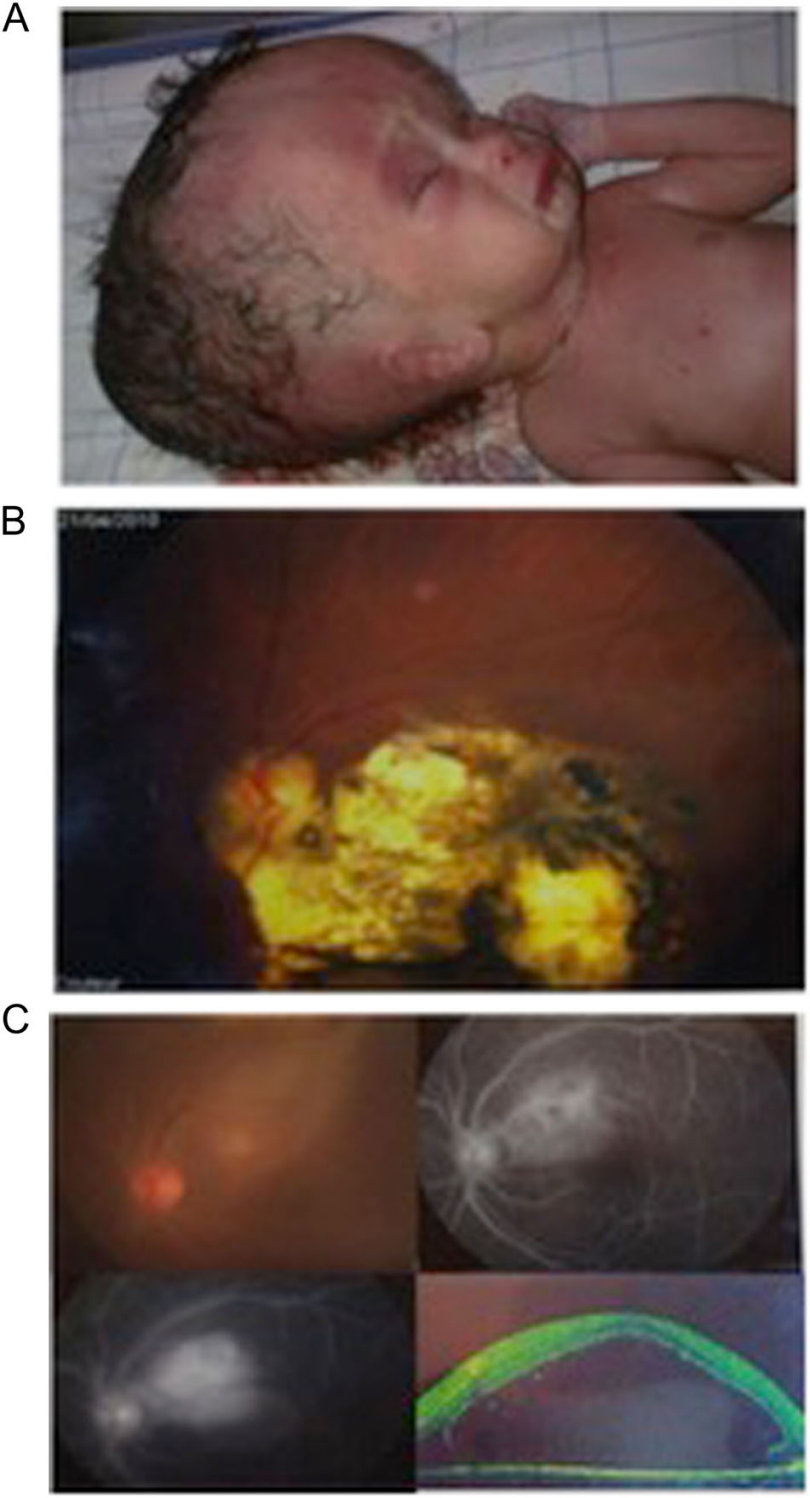
Congenital toxoplasmosis in developing countries. Manifestations of ocular disease are common in congenital toxoplasmosis. However, the disease can be quite severe, and patients from Morocco, shown here, demonstrate significant severe cases. **a** Severe hydrocephalus can occur due to inflammation preventing resorption of cerebrospinal fluid (CSF) or obstructing flow of cerebrospinal fluid (CSF). This child was admitted to the Hôpital des Spécialités in Rabat, Morocco, and unfortunately did not survive due to the severity of his illness. **b** Ocular involvement is also common in congenital toxoplasmosis. Substantial scarring of the retina is also possible, and disruption of the retina can lead to loss of sight. **c** Progression of an ocular lesion in a congenitally infected 19-year-old patient. Ocular lesions progress without adequate treatment and can involve the formation of subretinal fluid and choroidal neovascular membranes (observed using optical coherence tomography)

**Fig. 3:**
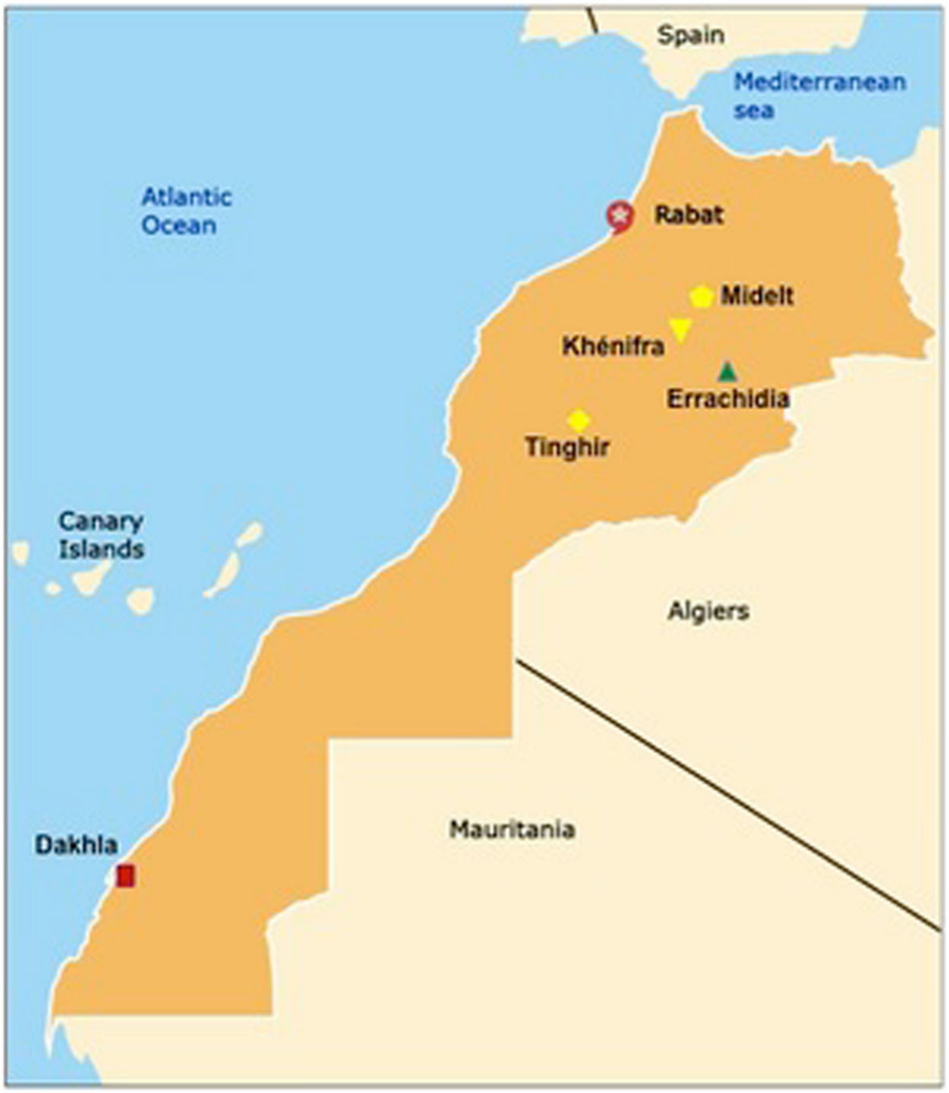
Congenital toxoplasmosis in Morocco. The geographic distribution of cases of congenital toxoplasmosis in a cohort of patients identified by Pr. Barkat are presented here and occurred across three distinct climate and cultural zones. The ocean region encompasses Rabat and Dakhla (indicated in red). The mountain region includes Khénifra, Midelt, and Tinghir (indicated in yellow), whereas the region of the Sahara includes Errachidia (indicated in green). Although the sample size is small, with only 21 cases of severe congenital toxoplasmosis, it is suggestive of the possibility that there is a geographic variation in the rates of this parasitic infection, potentially attributable to differences in climate or risk behaviors

Although *T. gondii* infection is prevalent nationally, there is still a significant population of seronegative pregnant women at risk for vertical transmission of the parasite. When identified, 7–24% of infected infants develop clinically significant eye disease. Severe congenital disease is not uncommon, and three patients, admitted to public hospitals in Rabat, demonstrate the severity of clinical disease in Morocco (Fig. 2). All these patients presented late and without antenatal or postnatal treatment. A collection of 21 congenitally infected infants occurred across three geographical regions (mountains, ocean, and Sahara) in an uneven distribution in 2015, none of whom received antenatal treatment. Despite the small sample size, this result suggests geographic variations in disease burden, highlighting the need for further data regarding disease prevalence and severity. Such studies are ongoing. The study presented in Table 1 and Fig. 3 is based on detection of *Toxoplasma*-specific serologies or individual clinician’s recognition of congenital cases. Additionally, molecular diagnostic tools have not been implemented in all public health laboratories, and information regarding contributing risk factors is limited. There is also a marked lack of information on genetic diversity of parasite strains in Morocco, in contrast to other studied countries. Clinical cases of congenital toxoplasmosis in Morocco are uncommon in the literature, highlighting the emerging nature of *T. gondii* infection in this part of the world. Although infection is clearly common, it has gone unrecognized by the global health establishment until very recently.

**Table 1:** Congenital toxoplasmosis in Morocco

Geographic region	Regional hospital centers	Number of births with *Toxoplasma* infection	Number of births in centers annually	Prevalence of severe congenital toxoplasmosis per 10,000 live births
Ocean	Dakhla	1	2179	4.6
	Rabat hospitals	15	38,000	3.9
Mountain	Khénifra	1	2300	4.3
	Midelt	1	2055	4.9
	Tinghir	1	1856	5.4
Sahara	Errachidia	2	2500	8

Babies confirmed to be born with congenital toxoplasmosis and exhibiting clinical signs of infection (chorioretinitis, hydrocephalus, and cerebral calcifications) were diagnosed by physicians and reported in 2015 by physicians across Morocco

The sum of these findings argues in favor of the implementation of a national surveillance system for this infection to provide valid prevalence data and follow-up of newborns at risk of symptomatic disease, as well as to ensure acquisition of data on the incidence of *Toxoplasma* infection during pregnancy in Morocco. Serologic screening during pregnancy, preventive educational programs, and early treatment should be implemented. These interventions would allow for systematic determination of rates of exposure to *T. gondii* in pregnant women and regular monitoring of uninfected, at-risk mothers, as in France, thereby detecting seroconversion and preventing morbidity and mortality in the unborn child caused by this emerging infectious disease.

### Colombia

Colombia, situated in northern South America, is a nation with extensive experience with congenital toxoplasmosis and a nominal GDP ranking of 39^46,55^. It is a tropical country with five different geographical regions: Andean mountains, Pacific coast, Caribbean coast, Amazon forest, and grassland plains. The seroprevalence of *T. gondii* antibodies is heterogenous between regions. Dry regions have lower frequencies of congenital toxoplasmosis (0–1 per 1000 newborns), and humid regions have a higher prevalence (2.5–3.8 per 1000 newborns)^23^. There is substantial evidence that parasitic strains in South America are more virulent than those found in France^56^. The disease presentation in Colombia is significantly worse than that in most European populations, and this difference has been associated with the parasite strain^57^. Neither testing nor treatment during pregnancy is provided for ~40% of pregnant women in Colombia, and this same situation is likely occurring in other countries in Latin America^23^. The high cost of laboratory testing (IgG, IgM, and avidity test to define the gestational age at which the pregnant woman’s conversion seroconversion occurred) and the lack of availability contributes to low screening rates. Of those mothers who do receive gestational screening, many are tested inadequately; they have only one test during pregnancy. With an initial negative IgG, which would merit further screening as the woman is still at risk for primary acquisition and vertical transmission, there is no further screening. This same inadequate screening is also occurring in Morocco. We encounter many cases of severely affected children, including with early mortality, in which the mother was tested only once during her first trimester without adequate follow-up. When public drinking water in Quindío was examined for *Toxoplasma* DNA, 60% of samples were positive^58^. This water contamination could explain upto 50% of the new cases and presents a significant, but solvable, challenge^59^. Waterborne outbreaks of *T. gondii* infection have been recognized in other South American countries and have resulted in mortality^4^. Therefore, implementing methods of surveillance and water treatment, along with recommendations regarding hygiene concerning water consumption, will likely positively impact rates of new infection in similar countries^60^.

**Fig. 4:**
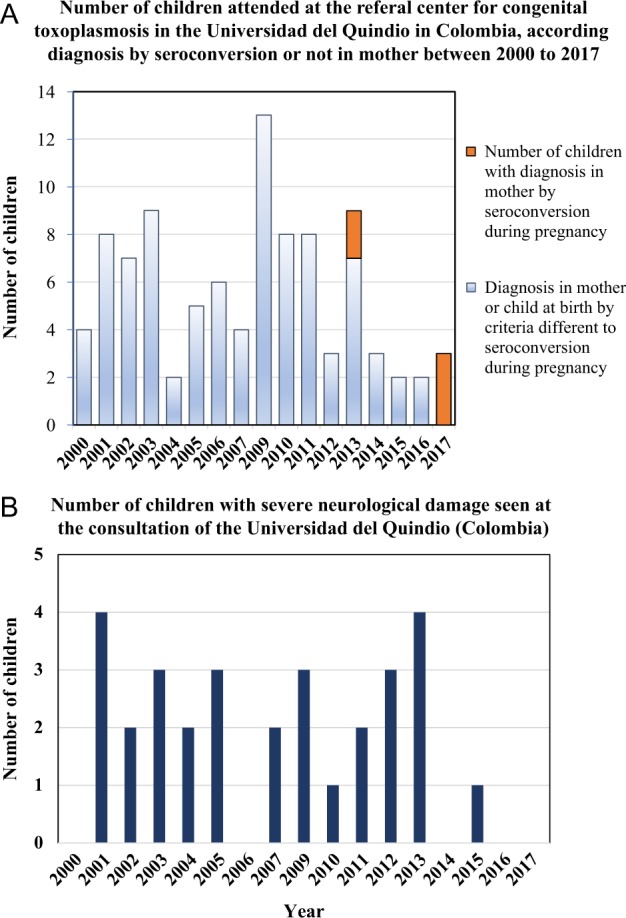
Evolution of criteria diagnosis and clinical presentation for congenital toxoplasmosis in the University of Quindio *Toxoplasma* consultation service before and after implementation of evidence-based guidelines in 2013. **a** Before 2013, when evidence-based guidelines for congenital toxoplasmosis were implemented, no cases based on seroconversion criteria presented for clinical consultation for congenital toxoplasmosis at the University of Quindio; after that date, three new cases were discovered by the monthly follow-up of mothers for the development of anti-*Toxoplasma* IgM antibodies. The congenital infection in children was confirmed by postnatal monthly serological follow-up. Children had prenatal treatment and had congenital infection that was asymptomatic. In 2017, only cases based on seroconversion diagnostic criteria were received, with none diagnosed at birth with severe disease, as is often the case in countries without systematic screening. **b** Before 2013, 2–6 cases of congenital toxoplasmosis with severe neurological damage (hydrocephalus or hydranencephaly) were identified annually at the Universidad del Quindio. After 2013, the only case that presented with severe neurological sequelae was an indigenous baby whose mother, for cultural reasons, did not participate in the prenatal screening program. There is a significant difference between the percentage of children seen with severe neurological damage in 2013 or earlier compared with after 2013 using Fisher’s exact test (29/65 = 44.6% vs. 1/10 = 10%, *p* = 0.04)

To face these public health challenges, in recent years, Colombia has invested in research on toxoplasmosis. The first formal guidelines (including GRADE recommendations, based on Cochrane Reviews, and economic evaluation) were developed in 2012 by Colombia´s Ministry of Health along with the Colombian Association of Infectious Diseases and the Colombian Association of Gynecologists for diagnosis and treatment of toxoplasmosis during pregnancy^60^. Official guidelines were launched in 2013 and included economic evaluation of one-time, three-time, or monthly testing during pregnancy. The analysis indicated that monthly testing can be cost-effective^61^. Positive benefits have become apparent after the publication and implementation of evidence-based guidelines; impacts have been observed at the congenital toxoplasmosis consultation service at the Universidad del Quindio, with marked increases in early diagnosis during pregnancy (Fig. 4a) and a reduction in severe cases (Fig. 4b).

### The United States

The United States is an ethnically and geographically diverse nation with a GDP ranking of 1^46^. Nonetheless, it also experiences challenges to proper identification and management of patients with congenital toxoplasmosis^62^. Estimates of seropositivity in the country as a whole are lower than those found in Morocco or Colombia, at approximately 13.2%, although there is clearly considerable regional variability. For example, seroprevalence is 50% in Amish women of childbearing age around Lancaster, Pennsylvania^6,41^. Additionally, one recent estimate of disease prevalence using insurance claims suggested that 2.4% of toxoplasmosis diagnosed in those with private insurance were congenital^62^. While this study suggested ~6900 cases of toxoplasmosis annually, it is likely an underestimate given the sampling bias. Privately insured patients may have different risk factors or exposures than their uninsured or publicly insured counterparts.

 The overall seroprevalence in United States is just over 10%, but certain subpopulations have rates of seroprevalence in excess of 50%^6^. Estimation of the incidence and prevalence of congenital toxoplasmosis has been reported in the literature and corresponds, in large part, to areas where seroprevalence is high^1^. The prevalence varies significantly with local conditions, including patient demographics and climate, among others. One study found that certain newborn infants, including those with lower socioeconomic status and of Hispanic descent, were at risk for more severe clinical disease^45^. One study indicated a higher seroprevalence among non-Hispanic black patients^63^. A more recent study seemed to indicate that the age-adjusted seroprevalence is decreasing over time in the United States for unclear reasons, but disparities likely remain^41^. Additionally, there are epidemics of *Toxoplasma* infection that occur in the United States, although they often go unrecognized^6^. *Toxoplasma* infection also tends to occur in familial clusters in the United States^64^. It is likely that this pattern occurs in other countries, although evidence for this is lacking.

Congenital toxoplasmosis has been well studied, with a national center at the University of Chicago in the National Collaborative Chicago-Based Congenital Toxoplasmosis Study (NCCCTS) cohort. This work defined treatment regimens and feasibility, safety, and efficacy of continuous treatment in the first year of life in reducing symptoms and sequalae. Continuous treatment with pyrimethamine, sulfadiazine, and folinic acid was then adopted in Paris for pregnant women and infants. NCCCTS patients have been studied longitudinally at regular intervals beginning in 1981 and have been extensively characterized^45,65,66^. Longitudinal data are a particular strength of the US experience with congenital toxoplasmosis. Disease severity in the United States is variable, though the NCCCTS cohort includes many severely affected children^67^. These individuals likely represent the most severe part of a spectrum of disease at presentation. More severely affected children are more likely to come to medical attention and the NCCCTS cohort. The NCCCTS has worked collaboratively with the Remington National Reference Serologic Laboratory, the reference laboratory for confirmatory testing in the United States. Another cohort that has contributed substantively to the US approach to congenital toxoplasmosis is the New England Regional Newborn Screening Program in Massachusetts and New Hampshire^68^.

Some high-end obstetrical practices in the United States provide monthly gestational testing for *T. gondii* infection. Nonetheless, neither reporting nor gestational screening is nationally mandated, presenting challenges to proper identification of disease burden in the United States. Gestational screening is expensive at present, with the American Academy of Pediatrics suggesting that routine screening may be unrealistic secondary to cost^69^. Furthermore, in the fragmented US healthcare system, uninsured patients often lack adequate access for even legally mandated testing. The advent of novel, more economical point-of-care screening modalities likely will revolutionize testing during gestation^70^. This will address one of the primary arguments against monthly gestational screening at present. For pregnant women identified as being acutely infected with *T. gondii*, medications for treatment are more widely available, in contrast to Morocco. However, changes within the pharmaceutical industry in recent years have presented new challenges to patients obtaining appropriate therapy.

### France

**Fig. 5:**
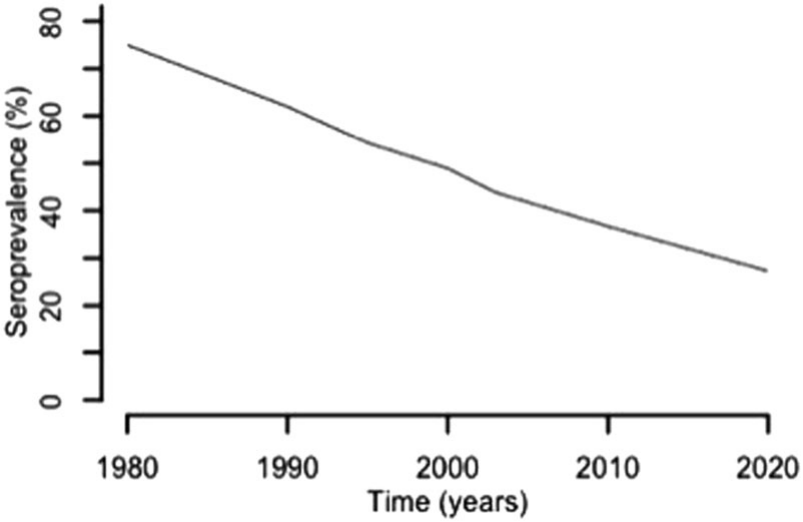
Decreasing seroprevalence in France over time. According to the French data collected over the last several decades, the seroprevalence for antibodies specific to *T. gondii* has decreased markedly^72^. In this graph, the seroprevalence data for 30-year-old women decreased over time, which is likely due to changes in risk behaviors influenced by educational programs as well as gestational screening. This downward trend in frequency is projected to decrease even further in the coming years. Decreasing disease severity in congenitally infected infants over this period has also been noted

France is a developed country with a GDP ranking of 6^46^. In many ways, the French experience with congenital toxoplasmosis represents an ideal to which others might aspire. Mandatory gestational screening for toxoplasmosis started in France in the late 1970s, became monthly in 1992 and is reimbursed by social security^71^. The French prevention program recommends monthly serological screening of susceptible women during pregnancy and information about hygiene and dietary recommendations. The seroprevalence of toxoplasmosis was approximately 43.8% in 2003 and decreased over several years due to improved hygiene conditions in meat-production and food preparation, including deep freezing^72^. A recent model-based estimation predicted that the incidence and prevalence of seropositivity in women aged 30 years for 2020 will be 1.6/1000 and 27%, respectively^72^. This decreasing seroprevalence over the last several decades is illustrated in Fig. 5.

Early diagnosis of congenital toxoplasmosis and treatment during pregnancy have proven to decrease transmission rates and improve clinical outcomes in affected children^9^. Another helpful approach in France is primary prevention of maternal infection. Physicians, obstetricians, and midwives are involved in patient education to reduce exposure to oocysts and tissue cysts. Risk factors are correlated with French food habits^53^. In addition to prevention and early detection of the disease, the management of congenital toxoplasmosis in France focuses on antenatal treatment. The French experience demonstrated the effectiveness of treatment of recently seroconverting, infected pregnant women in reducing rates of vertical transmission and congenital disease severity^9^. Depending on the gestational age at the time of infection, the infected mother is immediately treated with spiramycin and undergoes amniocentesis and fetal ultrasound. If amniotic fluid PCR is positive for *Toxoplasma* infection, treatment with pyrimethamine, folinic acid, and sulfadiazine or sulfadoxine should be initiated^73^. Infection is biologically confirmed after birth, and infected newborns are treated. The combination of pyrimethamine–sulfadoxine and folinic acid is used after the infant is 3 months old in southern France. In Paris, treatment is continuous pyrimethamine with folinic acid and sulfadiazine.

In France, more than 80% of human congenital toxoplasmosis is attributable to type-II *T. gondii* strains, characterized by slower growth and decreased virulence in mice^74,75^. Severe disease (often observed in Colombia, Morocco, or the United States) is less common in France. Rates of hydrocephalus in the NCCCTS were almost 31%, whereas they were markedly lower (0.3%) in France^76^. This finding likely, in part, reflects inadequate screening and delayed treatment, as most cases are detected at birth in the United States. Effective gestational screening programs and early prenatal treatment have reduced the prevalence and severity of congenital infection. French infrastructure permits monthly gestational screening followed by appropriate therapeutic interventions, in contrast to Morocco or Colombia, where even if screening occurs, adequate treatment and follow-up is often difficult to obtain.

## Discussion

Global variation exists in terms of the clinical characteristics of congenital infection with *T. gondii* and in the public health approaches to and circumstances surrounding management of this emerging infectious disease. With *T. gondii* causing disease outbreaks that lead to loss of sight, significant morbidity, and even mortality, optimizing these approaches would be of benefit. Morocco and Colombia face unique challenges to the appropriate management of the public health implications of toxoplasmosis. Morocco has a nationally mandated gestational screening but has not invested adequately in screening and treatment, and substantial risk is posed by oocyst contamination of soil. Moroccan research into parasite variation and infection epidemiology is limited, but preliminary data strongly suggest that *T. gondii* represents a significant and previously underrecognized public health burden in this North African country. Colombia, with virulent organisms and inadequate resources, is a nation where congenital toxoplasmosis continues to represent a significant risk to population health. Waterborne infection remains a substantial challenge in Colombia. Patients often remain untreated due to inadequate health infrastructure.

**Fig. 6:**
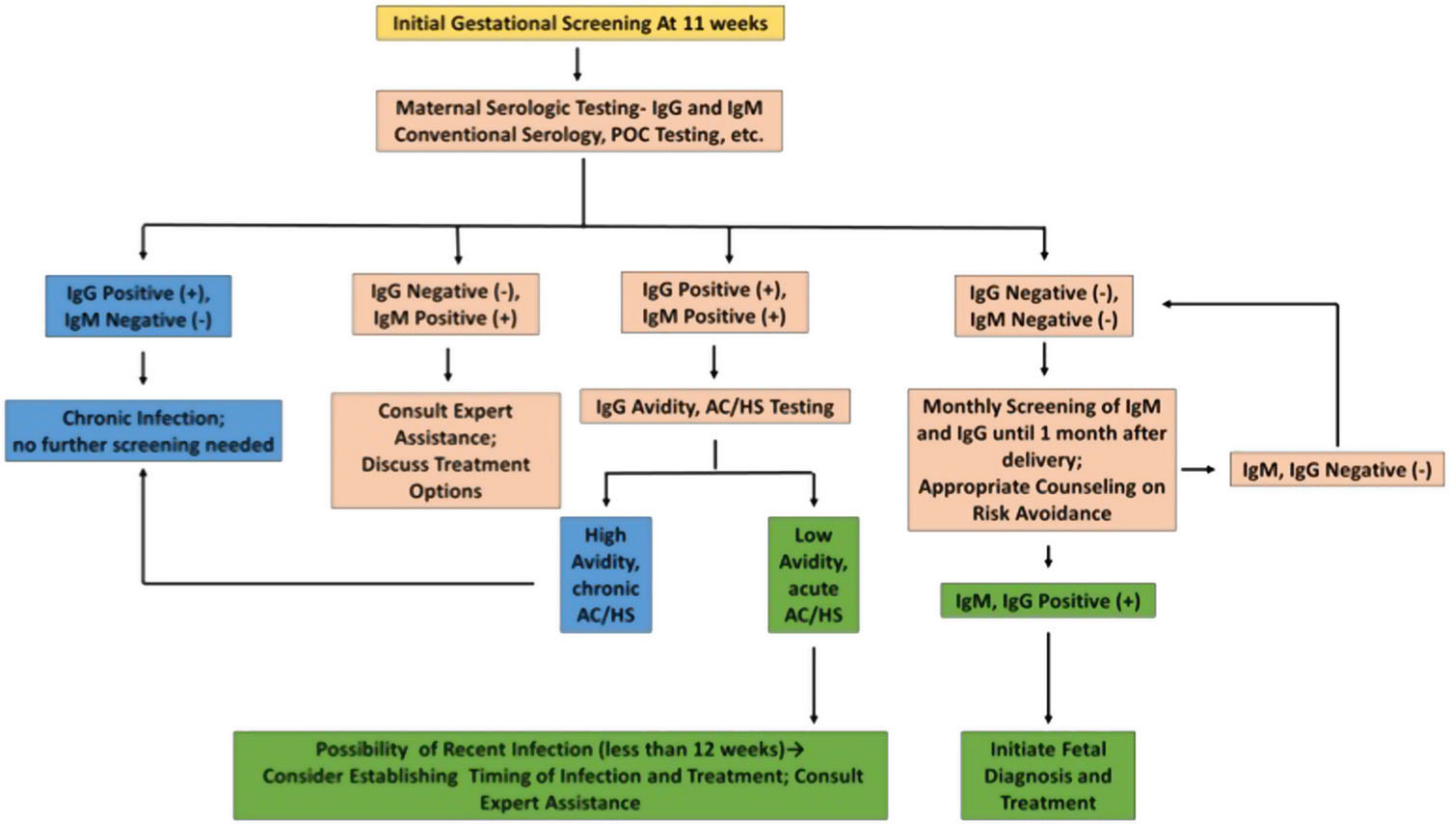
Initial gestational screening algorithm. A gestational screening algorithm, identifying those patients who ultimately require no treatment (blue), those who require additional testing (pink), and those for whom treatment should be strongly considered (green). This algorithm can be applied in any setting with basic laboratory capabilities, though avidity and AC/HS is more complex. In these settings, given the benign treatment profile of spiramycin, it might be appropriate to err on the side of treatment of these patients without further testing. The AC/HS test is a differential agglutination test using the ratio of two antigens present predominantly in early (AC) and late (HS) *T. gondii* infection to suggest the timing of infection. It is critical to note that this algorithm should only be utilized for pregnant women presenting for care during the specified time period. For those presenting at different times in gestation for care, testing and treatment protocols are more nuanced. Refer to work from the Palo Alto Reference Laboratory or papers on the management of congenital toxoplasmosis for further details regarding the screening protocol^8,13^

Developed nations are not immune to risks posed by toxoplasmosis. The United States represents a combination of parasite and host diversity, with substantial resources for management of this disease but inadequate allocation of these resources. The absence of mandatory gestational screening and a fragmented healthcare system with insufficient insurance coverage and access results in a poor understanding of the true scope of congenital toxoplasmosis there, and financial concerns limit access to screening. France, on the other hand, with mandatory gestational screening firmly ingrained and with standardized approaches to screening and treatment, has seen substantial reductions in disease prevalence and severity. France represents a potential gold standard for any country seeking to establish a program to address the public health challenge of congenital toxoplasmosis. The ideal approach to gestational screening, beginning at 11 weeks gestation, is in Fig. 6.

The natural question, then, is what drives differences between France and other analyzed nations in terms of congenital toxoplasmosis management and public health approaches? The answer is multifactorial but likely includes low prioritization for this parasitic infection given other health challenges, an unfamiliarity with the problem among health authorities, and differences in the cost–benefit ratio or availability of necessary resources. Given the clear benefits and mounting data favoring this approach, however, reallocation of resources must be considered, especially in the face of the epidemic nature of the parasite when outbreaks occur.

Optimal approaches combine appropriate gestational screening with an infrastructure that protects maternal–child health. Information regarding disease patterns, prevalence, and parasite and host genetic variation is needed. In an increasingly global society, infection with parasites of differing virulence is likely to occur in a variety of geographic locations. Modern individuals are highly mobile, and their pathogens travel with them. Shipment of agricultural products and migration patterns of birds also contribute to movement of strains globally. The role of animals in parasite transmission cannot be underestimated. A role for interruption of environmental transmission through vaccination programs in domesticated and feral cat populations may exist. A vaccine for cats was developed in the 1980s. A laboratory-attenuated strain of *T. gondii* was used to vaccinate cats and reduced disease transmission to pigs^77–79^. However, methods for effectively vaccinating feral and wild felids on a large scale thus far have remained elusive. There is no commercially available cat vaccine at this time. There are commercially available live vaccines for sheep, which were designed to reduce abortions associated with *T. gondii* infection^80,81^.

Additionally, therapeutic interventions must be made more widely available in a more economical fashion, such that those pregnant women and infants diagnosed with toxoplasmosis can be adequately treated. Possible approaches include the encouragement of more widely and easily distributed generic drugs, discovery and development of alternative medications, or vaccine development. This problem has many solutions on the horizon.

An optimal approach is multipronged. An educated population of pregnant women and physicians and others who provide care is an important first step. Reference centers can provide expertise in the sometimes-nuanced interpretation of serologic tests. Once a diagnosis is established, expertise in treatment is imperative, as well as the availability of medication and knowledge of its proper use. While no RCTs comparing screening and antenatal treatment to placebo exist, the preponderance of non-RCT evidence clearly identifies benefit. Given the availability of this data, establishing an RCT lacks equipoise. In our review of robust properly controlled, careful studies to date, we find that there is efficacy and reduction of suffering without significant harm with treatment, and screening can be accomplished easily and without harm. Treatment for gestational toxoplasmosis is not different in that regard from that of other congenital infections. Prusa et al. proved the favorable broad economic benefit of early identification and treatment of prenatal screening using real-world empirical data^12^. Benefits at individual and national levels have been noted in France and Austria. When some other countries have assessed available data on screening and antenatal treatment, their policy makers have prioritized their investments and policies concerning toxoplasmosis differently from those in France, Germany, Austria, Brazil, Uruguay, Panama, and Slovenia, which all provide screening. This screening is in countries with higher and lower incidence. Different countries set their own healthcare priorities. Robust point-of-care testing, with high sensitivity and specificity and low costs, along with multiplexed testing for other congenital infections that are also treatable, including syphilis, hepatitis B, and HIV, currently in development, will further facilitate screening programs^70,82,83^. These measures will save lives, prevent expenditures, and reduce suffering, in addition to providing substantial spillover benefits for maternal, child, and family health^70^.

Adequate reporting systems to understand disease burden and outcomes are also necessary. Once the epidemiology is understood, the implementation of primary prevention measures will improve public health. Water filtration, changes in dietary habits, and hand-washing are also valuable. The sum-total of these interventions serves as a paradigm for creating systems to optimize public health.

Toxoplasmosis harms human health directly and harms development and economic growth. The estimated total cost of *T. gondii* infections in the United States is approximately $3.3 billion USD annually, encompassing costs associated with spontaneous abortions, birth defects, and special healthcare costs^84,85^. With more than one-third of the world’s population chronically infected and the health implications of chronic infection across time only partially understood, these costs might be an underestimate of the financial and societal burdens of *T. gondii* infection^86^. The cost to developing countries will continue to increase if cases continue to increase and robust public health interventions are not implemented. Robust point-of-care testing with high sensitivity and specificity and low costs exists or is in development^70,82,83^. In between first trimester and end-of-gestation testing, *Toxoplasma* serology can be checked with newly developed, rapid POC tests, which are tractable, easy to use, and economical. It has been demonstrated that an LDBIO POC test, developed in France, is highly sensitive, specific, and low cost at $8 USD per test^70,82,83^. Additionally, new testing is in development to evaluate the role of testing saliva and whole blood, which will continue to revolutionize testing and make it more readily available for patients. Among the greatest challenges going forward will be the development of effective education programs for physicians and health officials in local communities, as well as ensuring that appropriate treatment and follow-up is available for affected patients. Efforts have been made to increase the availability of this information via educational pamphlets in a variety of languages (French, Spanish, Arabic, Tamazight (language of the Berber nomads) for the population in Morocco and Colombia).

These measures have the potential to save lives, prevent expenditures, and reduce suffering, in addition to spillover benefits for maternal and child health^70^.

Primary prevention programs, focused on the education of women to avoid potential parasitic exposures, may be of value and should be implemented alongside any prenatal screening program. Currently available evidence suggests a small benefit but is limited by poor study retention, so conclusions regarding efficacy are unclear^87^. Given the limited risks to these interventions, it would be sensible that they are a component of any program addressing congenital toxoplasmosis. Educational programs and public health interventions to improve hygiene and reduce risk behaviors have possible benefits beyond affected babies and their mothers, potentially reducing the burden of infection overall and thereby reducing reactivation ocular disease and neurological and disseminated disease in immunocompromised patients.

Some countries have prioritized their investments and policies concerning toxoplasmosis differently than others, choosing not to implement systematic screening. However, while decisions on national health policy must be matched to the needs and resources of individual countries, the existing evidence, as outlined here, strongly favors gestational screening and treatment of acutely infected mothers as well as robust public health interventions to address the ongoing threat of this emerging infectious disease. These interventions have been demonstrated to prevent morbidity and mortality, to reduce human suffering, and to be cost-saving, which is consonant with the ultimate goals of public health programs.
